# Anti-Coagulation During Pregnancy in Women with Mechanical Heart Valves: A Prospective Study 

**Published:** 2011-03-21

**Authors:** Amir Jamshid Khamoushi, Fahimeh Kashfi, Saeid Hosseini, Ali Reza Alizadeh Ghavidel, Niloufar Samiei, Mehdi Haddadzadeh

**Affiliations:** 1 Heart Valve Research Center, Shaheed Rajaei Cardiovascular Medical and Research Center, Tehran University of Medical Science, Tehran, Iran; 2 Epidemiology and Reproductive Health Department, Royan Institute for Reproductive Biomedicine, ACECR, Tehran, Iran; 3 Cardiac Surgery Department, Afshar Hospital, Yazd, Iran

**Keywords:** Mechanical Heart Valve, Anticoagulant, Heparin, Warfarin, Pregnancy

## Abstract

**Background:**

Pregnancy is associated with a hypercoagulable state, therefore the optimal anticoagulants for potential use in pregnant women with prosthetic heart valves are controversial. The aim of this study is to investigate the effect of anticoagulants on pregnancy outcomes and their potential risks in pregnant women with mechanical heart valves.

**Materials and Methods:**

In this prospective cohort study, we followed 44 women with 49 pregnancies who had mechanical heart valves from September 2002 to September 2007. A total of 38 patients took warfarin throughout their pregnancies (group A). In 11 patients, warfarin was changed to heparin during the first trimester and then again to warfarin during 12thto 36thweeks of gestational age (group B). All women took warfarin from 36thweeks of gestational age until delivery.

**Results:**

In group A, there were 22 live births (57.9%), 15 abortions (39.5%) and 1 maternal death (2.6%). In group B, there were seven live births (63.6%), three spontaneous abortions (27.3%) and one intra-uterine fetal death (9.1%). There was no significant difference in live birth rate between the two groups (p=0.24). Thirty-three pregnancies (86.8%) in group A and five pregnancies (45.4%) in group B had no maternal complications (p=0.004). The difference in pregnancy complications between both groups was significant (p<0.001)

**Conclusion:**

The present study shows that low dose warfarin (5 mg/day or less) may be safe during the first trimester of pregnancy. Maternal adverse events are low when pregnant women with mechanical heart valves remain on a warfarin regimen. The risk of embryopathy does not necessarily increase.

## Introduction

In pregnant women with mechanical heart valves, the frequency of valve thrombosis increases due to pregnancy-related hyper-coagulability. Therefore, effective anticoagulation is critical in these patients during pregnancy. Despite numerous research, the optimal anticoagulation therapy during pregnancy remains a controversial issue in the field of obstetrics. Warfarin and heparin can be used during pregnancy as anticoagulants in pregnant women with mechanical heart valves, however the potential maternal and fetal side effects associated with these medications pose challenges (1, 2).

Warfarin provides effective protection against thrombo-embolism, but its use in pregnancy is associated with an augmented rate of abortion and the risk of warfarin-induced embryopathy (3, 4). Warfarin is a teratogenic risk because of its ability to cross the placental barrier, particularly during early gestational age (1). First trimester complications of warfarin include: spontaneous abortion, prematurity, fetal deformity, stillbirth, retro-placental hemorrhage and intracranial hemorrhage. Warfarin embryopathy consists of bone and cartilage abnormalities, nasal hyperplasia, optic atrophy, blindness, mental retardation and seizures (4). More recent reports on the dose dependent effects of warfarin indicate safe administration during pregnancy if adequate anticoagulation can be achieved at doses of 5 mg or less (5, 6).

Unfractionated heparin (UFH) provides an alternative therapy that avoids fetal side effects; however, the use of UFH is associated with increased maternal thrombo-embolic and bleeding complications (1, 7, 8).

Low molecular weight heparin (LMWH) may be more advantageous than UFH and appears a good alternative (9), however scant information has been published on the use of LMWH in pregnant women with prosthetic heart valves (2).

There are several studies documenting the impact of mechanical heart valve replacement and anticoagulation therapy on pregnancy outcomes and risks, but most are retrospective. To date, no prospective study has been performed on Iranian women in this regard. Therefore, we designed a prospective research study to determine both maternal and fetal outcomes of pregnancy in women with prosthetic heart valves over a five year period in Rajaee Heart Hospital, as one of the cardiac surgery referral centers in Iran.

## Materials and Methods

In this prospective study, 49 pregnancies in 44 women with prosthetic heart valves were followed between 2002 and 2007 in the Department of Cardiac Surgery, Rajaee Heart Hospital, Tehran, Iran.

The Ethics Committee of Rajaee Heart Hospital approved this study. Participating patients signed informed consent forms. All patients were visited during the first trimester of their pregnancies. Pregnant women referred during their second or third trimesters were excluded from the study. Patients’ clinical and socioeconomic conditions determined their anticoagulant regimens. When the patient refused the recommended anticoagulation therapy, an alternative regimen was started. Thus, we had two study groups according to the anticoagulation regimen. A total of 38 patients were on warfarin (Orion Pharma, Finland) throughout their pregnancy (group A). The remaining 11 patients (group B) had iv injections of UFH (Rotex Medica, Germany) during the first trimester (6th-12th week), after which they received warfarin until the 36th week of gestation followed by heparin for the last two weeks of pregnancy. Both groups received heparin at the time of delivery. During warfarin treatment, the international normalized ratio (I.N.R.) was checked routinely and kept between 2.0 to 3.5, as needed. During heparin treatment, the activated partial thromboplastin time (aPTT) was maintained at twice the control level. All patients underwent periodic transthoracic echocardiography (TTE) in addition to transesophageal echocardiography (TEE) when needed during the follow up period. Pediatricians examined all newborns.

Spontaneous abortion was defined as fetal loss before 20 weeks of gestation. Fetal and maternal outcomes were evaluated. Fetal outcomes included abortion, live birth, intra uterine fetal death (IUFD), preterm labor, embryopathy and type of delivery; maternal outcomes included bleeding, valvular thrombosis and maternal death.

Statistical analysis was performed using SPSS version 13 software. Continuous variables were described as mean ± standard deviation (SD). Student’s t-test compared continuous variables. The Mann-Whitney rank-sum test compared medians when the normality test failed. Noncontinuous variables were compared by either the chi-square test or Fisher's exact test, as appropriate. A p value less than 0.05 was statistically significant.

## Results

The mean age of studied women at time of pregnancy was 29.8 ± 5.3 years (30.28 ± 5.2 in group A and 28.09 ± 3.9 in group B). There were 36 pregnancies in women with mitral valve replacement, 8 in women with aortic valve replacement and 5 in those with both aortic and mitral valve replacement (Table 1). None of pregnancies were twins. Five women became pregnant twice during the study duration (2002-2007).

**Table 1:** Type of surgeries in both groups


Variable	Group A (warfarin)	Group B(heparin)

**MVR**	28 (73.7%)	8 (72.7%)
**AVR**	5 (13.2%)	3 (27.3%)
**MVR and AVR**	5 (13.2%)	-


MVR: Mitral valve replacement AVR: Aortic valve replacement

Overall, 59% of pregnancies resulted in live births while 36.7% aborted. As shown in table 2, group A had 22 live births (57.9%), 15 abortions (39.5%), 1 IUFD (2.6%) and a maternal death due to acute thrombosis and prosthetic valve dysfunction in the delivery room. In group B, there were 7 live births (63.6%), 3 spontaneous abortions (27.3%) and 1 IUFD (9.1%). Live birth rates were similar between the two study groups (p=0.24).

There was no warfarin embryopathy in this study. After birth, during long term follow up of mothers and their babies, no obvious malformations were found in the infants.

Nine patients had vaginal delivery while cesarean section was performed in 20 patients. Abnormal post-partum hemorrhage was not seen in any woman. Twenty out of 49 pregnancies (40.8%) resulted in either abortion or IUFD. Despite the high rate of abortion in studied women, particularly in group A, we did not investigate the causes of spontaneous abortion. Both groups A and B had one stillbirth each (p=0.25).

Three infants were born prematurely, two in group A and one in group B.

Overall the low birth weight rate was 21% in our study (6 out of 29 cases). Although this rate was higher in group A (23% versus 14%), however this difference was not statistically significant (p>0.05).

**Table 2:** Fetal outcome in 49 pregnancies


Pregnancy outcome		Group A (warfarin)n=38	Group B(heparin)n=11	P value

**Abortion**		15 (39.5%)	3 (27.3%)	0.35
	**Spontaneous**	9 (23.7%)	3 (27.3%)	
	**Therapeutic**			
**Live births**		22 (57.9%)	7 (63.6%)	0.5
	**NVD**	7 (18.4%)	2 (18.2%)	
	**CS**	15 (39.5%)	5 (45.5%)	
**IUFD**		1 (2.6%)	1 (9.1%)	0.4


NVD: Normal vaginal delivery CS: Cesarean section

Prevalence of valve-related thrombosis and prosthetic valve dysfunction (PVD) was significantly higher in group B (45.4% versus 2.6%; p<0.001). Valve-related complications were seen in 13.2% of group A cases and 54.6% from group B (p=0.004; Table 3).

**Table 3:** Maternal complications in 49 pregnancies


Variable	Group A(warfarin)n=38	Group B(heparin)n=11	P value

**No complications**	33 (86.8%)	5 (45.4%)	0.004
**Maternal death**	1 (2.6%)	-	0.77
**Prosthetic valve dysfunction in first trimester**	1 (2.6%)	5 (45.4%)	0.001
**Prosthetic valve dysfunction in third trimester or after delivery**	3 (7.9%)	1 (9.2%)	0.65


Six patients in the heparin group had valve thrombosis and underwent additional surgery. Five of the six had severe heart failure as a result of valve thrombosis during the first trimester, while one had thrombosis at the end of the third trimester and underwent surgery during the postpartum period. Only three (50%) of these pregnancies with valve thrombosis in group B resulted in live births. In group A, valve thrombosis occurred after delivary in 2 patients (5.3%) among 4 patients with valve thrombosis.

Table 4 shows the pregnancy outcome according to warfarin dosage. Most abortions occurred in the warfarin group at doses greater than 5 mg daily (p=0.016; Table 4).

**Table 4:** Pregnancy outcome according to warfarin dosage in group A


Outcome	Warfarin≤5 mgn=29	Warfarin>5 mgn=9	P value

**Abortion**	8 (27.6%)	7 (77.8%)	0.016
**Live birth**	20 (69.0%)	2 (22.2%)	0.021
**Maternal death**	1 (3.4%)	-	0.76


## Discussion

The combination of heart disease and pregnancy can present a challenge to the physician caring for both the mother and fetus (10). Pregnancy after mechanical heart valve replacement requires strict control of coagulation. Special attention should be paid to the occurrence of complications during anticoagulation therapy (11). The risk of thromboembolism, miscarriage and premature birth seems to be higher in patients with prosthetic heart valves that require anticoagulation (4).

Warfarin crosses the placental barrier and its use by the mother may result in an increased incidence of fetal mortality and morbidity. Treatment with this agent during pregnancy is associated with a high spontaneous abortion rate that ranges between 16.2% and 44% (4, 12).

The use of heparin presents an attractive alternative to warfarin because it does not cross the placenta (13).

In the present study, the overall abortion rate was 39.5% in the warfarin group of which 15.8% were therapeutic abortions. The heparin group had more spontaneous abortions (27.3%) compared to the warfarin group (23.7%), although this difference was not significant. This finding contrasted studies by Nassar et al. (8), Geelani et al. (14), Cotrufo et al. (15) and Al-lawati et al. (16) who noted more spontaneous abortions in the warfarin group, although only Nassar et al. (8) found this difference to be significant. Our result was consistent with Akhtar et al. (17) although in our study the increased abortion rate in the heparin group was not significant, whereas Akhtar et al. showed a significantly higher spontaneous abortion rate in the heparin group. Salazar and colleagues have reported a 37.5% incidence of abortions in a series of patients treated with subcutaneous heparin during the first trimester of pregnancy. These high abortion rates in both groups could be explained by placental hemorrhage, which may occur during effective anticoagulation with either warfarin or heparin (13).

The rate of healthy babies born by these mothers was 57.9% in group A and 63.6% in group B, which is similar to results reported by Nassar et al. (8) and Kim et al. (18).

In this study, there was no warfarin-induced fetal malformation, and is similar to other studies by Vural et al. (19), Geelani et al. (14) and Al lawati et al. (16). It can be suggested that the teratogenic effects of warfarin during the first trimester may be overstimated, therefore the effects of switching to heparin must be studied further with additional larger studies.

In the present study, thromboembolic complications were significantly more frequent in the heparin group (45.4%) compared to the warfarin group (2.6%; p=0. 001). Although the live birth rate was higher in the heparin group, the use of heparin resulted in a higher spontaneous abortion rate. It seems the use of subcutaneous UFH during pregnancy raises the concern of an increased incidence of maternal risk in the form of valve thrombosis without any significant reduction in the incidence of spontaneous abortion compared with warfarin (2, 13). We found significantly more valve thrombosis and second surgeries in the heparin group. Another challenging issue in this regard is the dose of warfarin used in pregnant women with mechanical heart valves. In the present study, warfarin at doses over 5 mg daily resulted in a significantly higher abortion rate and lowered live birth rate. The only woman who expired in the warfarin group was treated with a low dose of warfarin. In a previous study in our center, Khamooshi et al. have shown that warfarin is an effective anticoagulant in pregnant women with mechanical valves, but it results in significant fetal loss at doses over 5 mg daily (20). Vitale and colleagues have shown a close relationship between fetal complications and warfarin dosage (5, 6). We previously described a case report regarding the safety of low dose warfarin during pregnancy in a woman with two mechanical heart valves who became pregnant by the intracytoplasmic sperm injection (ICSI) method (21).

Cotrufo and colleagues described the outcomes of 71 pregnancies in women with mechanical valve prostheses who were anticoagulated with warfarin for the entire duration of their pregnancies. Although there were no maternal deaths, or thromboembolic or hemorrhagic complications, the rates of miscarriage (32%), stillbirth (7%) and embryopathy (6%) increased (15). It seemed the risk of complications was higher when the mean daily dose of warfarin exceeded 5 mg (15, 20).

One of present study’s limitations is the relatively low sample size that prevented reaching a definite conclusion.

## Conclusion

It can be suggested that if patients on warfarin switch to heparin no embryopathy occurs, however there would be a risk of maternal complications. In addition, this study confirms previous studies that low dose warfarin (≤5 mg/day) during pregnancy is almost safe, with minimal feto-maternal complications. Additional prospective multicentric studies with more cases are recomended.
